# Association between Lifetime Tobacco Use and Alcohol Consumption Trajectories and Cardiovascular and Chronic Respiratory Diseases among Older People

**DOI:** 10.3390/ijerph182111275

**Published:** 2021-10-27

**Authors:** Ignacio Madero-Cabib, Claudia Bambs

**Affiliations:** 1 Departamento de Salud Pública, Instituto de Sociología, Pontificia Universidad Católica de Chile, Santiago 7820436, Chile; 2 Research Center Millennium Nucleus for the Study of the Life Course and Vulnerability (MLIV), Santiago 7820436, Chile; 3 Departamento de Salud Pública, Facultad de Medicina, Pontificia Universidad Católica de Chile, Santiago 8330077, Chile; cbambs@uc.cl; 4 Advanced Center for Chronic Diseases (ACCDiS), Facultad de Medicina, Pontificia Universidad Católica de Chile, Santiago 8330077, Chile

**Keywords:** tobacco use, alcohol consumption, chronic diseases, life course, older people, Chile

## Abstract

**Background:** We identify representative types of simultaneous tobacco use and alcohol consumption trajectories across the life course and estimate their association with cardiovascular and chronic respiratory diseases (CVDs and CRDs) among older people in Chile. **Methods:** We used data from a population-representative, face-to-face and longitudinal-retrospective survey focused on people aged 65–75 (N = 802). To reconstruct trajectory types, we employed weighted multichannel sequence analysis. Then, we estimated their associations with CVDs and CRDs through weighted logistic regression models. **Results:** Long-term exposure to tobacco use and alcohol consumption across life are associated with the highest CVD and CRD risks. Long-term nonsmokers and nondrinkers do not necessarily show the lowest CVDs and CRDs risks if these patterns are accompanied by health risk factors such as obesity or social disadvantages such as lower educational levels. Additionally, trajectories showing regular consumption in one domain but only in specific periods of life, whether early or late, while maintaining little or no consumption across life in the other domain, lead to lower CVDs or CRDs risks than trajectories indicating permanent consumption in both domains. **Conclusions:** A policy approach that considers CVDs and CRDs as conditions that strongly depend on previous individual experiences in diverse life domains can contribute to the improved design and evaluation of preventive strategies of tobacco use and alcohol consumption across the life course.

## 1. Introduction

Several epidemiological studies have demonstrated that regular tobacco use and harmful alcohol consumption are crucial determinants of cardiovascular diseases (CVDs) [1,2,3]. Although the majority of these findings come from cross-sectional analyses, increasing longitudinal evidence has shown that persistent patterns of tobacco and alcohol consumption influence CVDs [4,5,6]. The association between smoking patterns and chronic diseases and all-cause mortality is well documented [7], and longitudinal research has shown the health benefits of smoking cessation, particularly if it occurs early in life [8]. However, recent evidence has shown that those who quit smoking earlier in life and experience weight gain afterward are at higher risk of CVDs [2,9,10,11].

For decades, studies have reported a J-shaped association between alcohol consumption trajectories and the risk of CVDs, meaning that people who never drink and permanent heavy drinkers were more likely to develop CVDs [12,13,14,15,16]. Yet, recent evidence has revealed that those findings were subject to methodological bias due to the cross-sectional design of most studies and a lack of adequate adjustment by gender, educational level, ethnic background, and the type of alcoholic beverage measured, among others. Recent research has shown that drinking has either a nonsignificant or no protective effect on all-cause mortality and CVD outcomes, emphasizing that alcohol use, regardless of amount, leads to health loss across populations [17,18].

On the other hand, tobacco consumption is recognized as one of the main causal factors of chronic respiratory diseases (CRDs) [19]. Increasing evidence has also pointed out the effects of alcohol on lung health due to factors such as a higher risk of pneumonia, tuberculosis, respiratory syncytial virus infection, and acute respiratory distress syndrome among individuals with alcohol use disorders. Recent research has contributed to a better understanding of the pathophysiology of alcohol-induced lung injury and the effects of alcohol on lung immune response, which might lead to the development of CRDs [20].

To the best of our knowledge, no studies have examined the associations of simultaneous long-term smoking and alcohol consumption patterns with these chronic diseases. Besides, most evidence on the behavioral risk factors of CVDs and CRDs comes from studies in developed and high-income countries. However, analyses from developing countries remain scarce. Cardiovascular and chronic respiratory diseases are among the most prevalent non-communicable diseases worldwide [21,22] and are among the four main causes of premature death and disability in Latin America [22,23].

The main research question guiding this exploratory study is how simultaneous trajectories of tobacco use and alcohol consumption across the life course are associated with CVDs and CRDs among older people in Chile.

The main motivation to focus on simultaneous patterns of tobacco use and alcohol consumption and their relationship with CVD and CRD risks is that these two health behaviors frequently coexist: compared to the average population, regular tobacco users are four times more likely to be dependent on alcohol, and people with alcohol dependence are three times more likely to be smokers [24]. However, it is not clear, for example, whether the concomitant use of alcohol can increase the likelihood of CRDs or CVDs among smokers. In other words, our main motivation in this study is to explore whether health advantages in one domain (e.g., being a permanent nonsmoker) may offset health disadvantages in the other domain (e.g., being a permanent heavy alcohol drinker); conversely, disadvantages in both domains may interact, leading to higher health risks.

Chile is a Latin American developing country with nearly universal health coverage but unfortunately also with stark health inequalities between socioeconomic groups and also institutionalized inequities in the health system [25]. The latter has been identified, for example, in the performance of the system for elderly and non-elderly age groups, the elderly being those with fewer health needs covered and the least financially protected in case of health problems, particularly in the frame of the private health system [25].

In this country, noncommunicable diseases account for 85% of all deaths among adults [26]. According to the National Health Survey [27,28], 10.0% of people aged 65 and over have experienced CVDs, 74.2% of the population are overweight or obese (the highest among all OECD countries) [29], and 37.8% of men and 29.1% of women are current smokers. The proportion of users of illicit cigarettes is rather low (about 10.9%) [30]. Additionally, 11.7% of the Chilean population aged 15 or more are at risk of alcohol use disorders, reaching 20.5% among males [27]. Overall, Chile has one of the highest rates of alcohol use per capita in the Americas (9.3 L of pure alcohol per capita) and the prevalence of heavy episodic drinking is high (32% among drinkers from the general population 15+ years old, reaching to 52% among 15–19 male drinkers) [31].

In terms of health policies, Chile has implemented tobacco control regulations since 2005, such as protecting smoke-free environments and imposing marketing regulations and tax increases on tobacco products, which have led to a decline in smoking among the general population, particularly among the youth [32,33]. We also know that not consuming tobacco in this country is associated with higher health and education expenditures, especially among poor individuals [34]. However, one in three Chileans and 19% of people aged 60 and over continue to smoke cigarettes [35]. With respect to alcohol health policies, in 2014, Chile introduced a tax increase for alcoholic drinks, but only in 2021 is a marketing regulation regarding the advertising and sponsorship of alcohol products being implemented. Additionally, alcohol is cheap and easily available compared with other countries. Taken together, tobacco and alcohol policies still have wide room for improvement in Chile, as tobacco and alcohol consumption patterns are still among the top six risk factors of deaths among Chileans [32].

As discussed below, the findings of this exploratory research can provide important implications to guide improved health policies on tobacco and alcohol use through both individual-level strategies (e.g., for health professionals to discuss with patients the benefits of a life-course free of tobacco and alcohol) as well as population-level strategies (e.g., for policymakers to acknowledge the relevance of a life-course approach for the design and evaluation of preventive strategies that affect tobacco and alcohol consumption across the life span).

## 2. Materials and Methods

### 2.1. Data

We used data from the “Life Course and Vulnerability among Older People in Santiago, Chile” study, a population-representative, face-to-face, and longitudinal-retrospective survey. The survey was conducted by the first author of this study and aimed to collect retrospective annual information on behavioral risk factors and other dimensions of the life course for 802 people born between 1944 and 1954 (currently aged 65–75) living in 2019 in Santiago, Chile. The focus on this age range arises from the interest in this survey to also understand the labor force dynamics around the retirement age. The survey was conducted following the latest quality standards defined by the American Association for Public Opinion Research [36]. To be certain that our results were not affected by potential sample selection bias, the study sample was weighted to provide representative results for older adults aged 65–75 in the city of Santiago.

The process of data collection involved the use of life-history calendars that helped respondents remember and chronologically organize the various episodes during their lives, along with approximate dates of occurrence [37]. The literature on this field has shown that the retrospective recall of events is not a linear process, but a cognitive action involving three different mechanisms: first, the hierarchical order of life events (i.e., those more and less relevant for each person); second, the sequential order of events (i.e., which event happened first and which event happened afterward); and third, the parallel order of events (i.e., the interrelations between the occurrence of events in one domain and the occurrence of events in other domains) [38]. The life-history calendar used in this study addressed these three mechanisms of operation of the autobiographical memory by first, including a timeline that forced individuals to think retrospectively about their lives in a chronological way; second, by making individuals remember events in key domains (e.g., births, marriage, divorces, or widowhood) to then relate them to other events in the rest of the social domains, thereby increasing the accuracy of the reconstruction of the past; and third, by giving respondents the chance to add information to the calendar even if this meant going backward/forward in time.

Appendix A provides detailed information about the sampling frame, sample selection, measures during the pretest phase, cooperation, response and refusal rates, weighting strategy, and more description of the life-history calendar used to address the mechanisms involved in autobiographical memory.

### 2.2. Measures

#### 2.2.1. Tobacco Use and Alcohol Consumption Trajectories

We measured life trajectories in two dimensions: tobacco use and alcohol consumption. People were asked annually whether they had smoked and drunk alcohol occasionally, regularly, or not at all. With this information, we were able to build a life-history dataset in which each individual was observed every year from birth until the time of the interview, repeatedly recording the same state for each dimension until there was a change in their status. To reconstruct tobacco use trajectories, the following categories were used: (1) “does not smoke”, (2) “smokes occasionally” (a few times a year), and (3) “smokes regularly” (daily). To reconstruct alcohol consumption trajectories, the following categories were used: (1) “does not drink alcohol”, (2) “drinks alcohol occasionally” (a few times a year), and (3) “drinks alcohol regularly” (at least once a week).

Exposure to smoking and alcohol consumption in the life-history calendars was based on frequency questions and did not include aspects such as the number of cigarettes or alcoholic drinks consumed per occasion and the presence of dependence symptoms. This is mainly because of the challenges involved in the retrospective recall of specific events across the life course in this type of retrospective questionnaire [36,37].

#### 2.2.2. CVDs and CRDs

To measure outcomes, we used the participants’ self-reports of whether they had ever been told by a medical doctor that they had CVDs or chronic lung diseases, such as chronic bronchitis or emphysema.

#### 2.2.3. Control Variables

All models were adjusted by age, gender, educational level, body mass index (BMI), physical activity, and frequency of consuming fruits and vegetables.

### 2.3. Statistical Analysis

To reconstruct the trajectory types in the two domains of interest, we used multichannel sequence analysis (MCSA) [39], an extension of traditional sequence analysis [40]. MCSA is a longitudinal statistical technique useful for exploratory studies in which the main aim is to understand unknown long-term patterns in different life domains through highly illustrative visualizations and a holistic perspective, focusing on the whole trajectory rather than on specific transitions that compose a trajectory [40]. MCSA simultaneously estimates typical life trajectories in more than one domain, relying on the analysis of similarities between all possible pairs of individual trajectories in those domains.

In our study, we considered two individuals as experiencing similar trajectories if both faced similar tobacco use and alcohol consumption statuses across time in a similar order and at similar time points. A pairwise distance matrix summarizes the “distance” between the individual sequences, which represents the number of modifications (“costs”) that are needed to make trajectories in two domains of one individual exactly like the trajectories in the same two domains of another individual. To estimate distances, we used optimal matching analysis and followed the recommendations to use a substitution cost of 2 and an indel cost of 1 [41].

**Figure 1:**
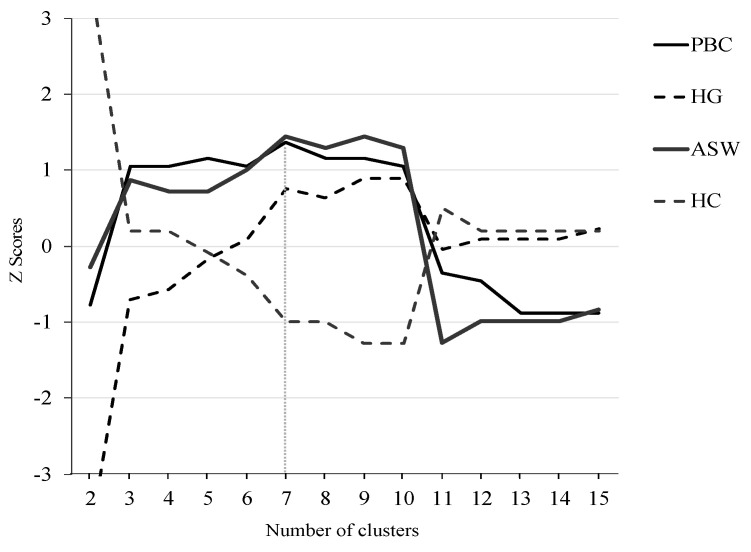
Cluster cut-off criteria. Note: All measures are standardized. PBC: Point Biseral Correlation. HG: Hubert’s Gamma. ASW: Average Silhouette width. HC: Hubert’s C coefficient.

Over the distance matrix, hierarchical agglomerative clustering can be conducted using the Ward algorithm to group individuals who experience similar trajectories into representative types. Then, a robust cluster solution is selected to summarize the representative trajectory types, followed by individuals in the tobacco use and alcohol consumption domains. For this purpose, we employed four cluster selection criteria: average silhouette width, point biserial correlation, Hubert’s gamma, and Hubert’s C. Based on these selection criteria (see Figure 1), our final solution for the tobacco and alcohol consumption trajectories had seven representative types.

The identified tobacco and alcohol trajectory types became the main independent variable in two sets of weighted logistic regression models: one predicting CVDs and another predicting CRDs. We estimated all models in R using the TraMineR package [42] for MCSA and the survey package for weighted surveys.

## 3. Results

### 3.1. Weighted Univariate Descriptives

Table 1 shows the weighted distribution of the sample across the dependent variables and the sociodemographic and health risk variables. The majority of the sample were women and had relatively low levels of education. The average age was around 70 years. About a third were obese and engaged in physical activity once a week or less. The participants’ diets included high levels of fruits and vegetables. Of the sample, 17.34% had been diagnosed with CVDs by a medical doctor, and 6.16% had been diagnosed with CRDs.

### 3.2. Tobacco Use and Alcohol Consumption Trajectories

**Figure 2:**
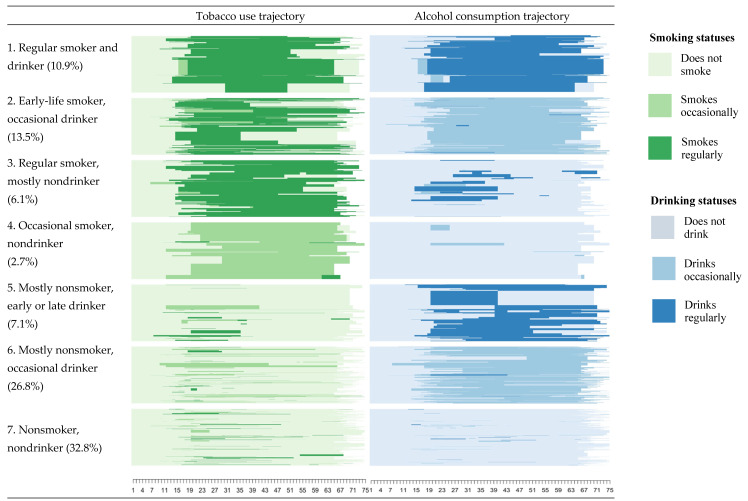
Sequence index plots of seven types of weighted lifetime tobacco and alcohol use trajectories.

Figure 2 shows sequence index plots for the seven types of tobacco use and alcohol consumption trajectories. Sequence index plots display in the *x*-axis the age, and in the y axis, one line for each trajectory of each individual from age 1 to 65–75 (depending on the respondent’s age at the interview), where a change of color symbolizes a change of status. All percentages displayed are weighted. Table 2 shows the weighted distribution of CVDs, CRDs, gender, education, BMI, physical activity, and frequency of consuming fruits and vegetables by trajectory type.

Type 1, “regular smoker and drinker”, represents 10.9% of the study sample and is characterized by people who smoked regularly during long periods of adulthood and drank from age 20 until late life. It has the highest average prevalence of CVDs (53.1%) and CRDs (12.5%). People of this type are relatively more educated (only 13.0% only have a primary education) and mostly male (87.4%).

Type 2 (“early-life smoker, occasional drinker”, 13.5%) is composed of regular tobacco users, especially in late adolescence and early adulthood (ages 15–35), and occasional alcohol users across the entire life course. Figure 2 shows that many individuals in this trajectory stopped smoking in mid-to-late adulthood (ages 35–55). Type 2 has the lowest prevalence of CVDs (5.6%) but the second highest prevalence of CRDs (11.2%). Almost a third are obese (31.7%), and 39.2% have only a primary education.

Type 3 (“regular smoker, mostly nondrinker”, 6.1%) is characterized by regular smokers who started using tobacco roughly from age 15-onward but did not drink for most of their lives (only in short periods of adulthood). Among them, 11.7% and 6.8% reported having been diagnosed with CVDs and CRDs, respectively. The majority (71.8%) of this group do physical activity once a week or less, and 30.9% consume fruits and vegetables six times a week or less.

Type 4 (“occasional smoker, nondrinker”) represents a small proportion of people (2.7%) who occasionally used tobacco across their life course and had almost no alcohol consumption. Individuals in this group have a relatively low prevalence of CVDs (9.9%) and a slightly lower prevalence of CRDs (5.0%) than the sample average (6.2%). The group is composed mostly of women (80.0%) and has a relatively low prevalence of obesity (14.9%).

Type 5 (“mostly nonsmoker, early or late drinker”, 7.1%) is characterized by individuals who smoked only during early adulthood and drank regularly either in early or late adulthood (see Figure 2). This type has the lowest prevalence of CVDs (4.0%) and CRDs (3.1%). The group is composed mostly of men (81.2%), and a large proportion have a tertiary education (44.7%). More than half (58.9%) engage in physical activity more than once a week.

Type 6 (“mostly nonsmoker, occasional drinker”, 26.8%) and Type 7 (“nonsmoker, nondrinker”, 32.8%) are composed of people who did not smoke for most of their lives and drank occasionally from around age 20 (Type 6) or did not drink (Type 7). While both types have a low prevalence of CRDs (3.3% and 4.9%, respectively), their prevalence of CVDs (20.3% for Type 6 and 12.4% for Type 7) is similar to that of the sample average (17.3%). Types 6 and 7 are composed of large proportions of women (60.0% and 76.6%). Less than half have a primary education (37.8% and 47.6%) and are obese (31.0% and 43.0%).

### 3.3. Tobacco–Alcohol Trajectories and CVDs and CRDs among Older People

Table 3 shows two separate weighted logistic regression models indicating the probability of experiencing CVDs and CRDs. The relative risk ratios (RRRs), average marginal effects (AMEs) specifically for the trajectory types, p values, and 95% confidence intervals are reported in the models. We used Type 1 (“regular smoker and drinker”) as the reference group for the dependent variable because it has the highest average prevalence of CVDs and CRDs. Compared to Type 1, all trajectory types have a negative association with both CVDs and CRDs.

In the model predicting CVDs, all trajectory types show statistically significant results. Although we expected Type 6 (“mostly nonsmoker, occasional drinker”, RRR = 0.24, AME = 0.30, *p* < 0.1) and Type 7 (“nonsmoker, nondrinker”, RRR = 0.12, AME = −0.30, *p* < 0.001) to indicate the most protective effects because of the low or nonexistent tobacco and alcohol consumption, we observed that Type 5 (“mostly nonsmoker, early or late drinker”, RRR = 0.03, AME = −0.47, *p* < 0.001) was the least likely to experience CVDs. Specifically, belonging to Type 5 reduced the probability of experiencing CVDs by 47 percentage points on average.

Finally, confirming the bivariate results, belonging to Type 2 (“early-life smoker, occasional drinker”) reduced the probability of having CVDs by 44 percentage points on average (AME = −0.44, *p* < 0.001) in comparison to Type 1.

In the model predicting CRDs, only the trajectory types indicating occasional or no tobacco use across life (Types 4, 5, 6, and 7) show statistically significant results. Compared to Type 1, the lower probabilities of experiencing CRDs are fairly similar across these trajectory types, which have AMEs ranging from 14 to 15 percentage points and RRRs ranging from 0.14 to 0.21. Therefore, the main finding of the logistic regression for CRDs is that regular tobacco use, regardless of whether it is only during specific periods in early life (Type 2) or persistent across the life course (Types 1 and 3), is associated with a high chance of experiencing CRDs. Also, while some people who occasionally used tobacco across the life course (Type 4) or consumed alcohol regularly in early or late life (Type 5) still have lower CRD risks compared to people in Type 1, these effects of Types 4 and 5 are possibly driven not by their tobacco and alcohol life trajectories but by the absence of other health risk factors, such as the relatively low proportion of obese individuals (14.9% and 19.0%, respectively) and those with only a primary education (22.4% and 25.0%, respectively).

## 4. Discussion

Our findings demonstrate that, first, patterns of long-term exposure to tobacco use and alcohol consumption across life (Type 1) are associated with the highest CVD and CRD risks.

Second, health advantages in both domains (i.e., being a nonsmoker and nondrinker across life [Type 7]) do not necessarily lead to the lowest CVD and CRD chances if these patterns are accompanied by other health risk factors, such as obesity, or social disadvantages, such as lower educational levels (as with Types 6 and 7). In a similar vein, the high presence of health-protective factors such as tertiary education, normal body weight, and engaging in weekly physical activity, at least in part, can counterbalance the CVD risk associated with regular alcohol consumption either in early or late adulthood (Type 5). Our findings are consistent with previous studies that show that combined risk lifestyle factors, including tobacco, alcohol consumption, low physical activity, and unhealthy diet have a substantial impact on total and cause-specific mortality [43,44,45]. This is important considering that it is known that individual lifestyle factors tend to cluster within a population and that interaction effects may exist among them, thus making the study of joint effects particularly informative for the purpose of diseases prevention and useful for policymakers in targeting local needs and priorities [46].

Third, the roles of the explored domains are not equally relevant to the chronic diseases analyzed. For example, when evaluating CRDs, disadvantages in the alcohol consumption domain (i.e., regular consumption in early or late life [Type 5] or occasional consumption across life [Type 6]) do not hinder advantages over CRDs in the tobacco use domain (i.e., being mostly a nonsmoker). Conversely, disadvantages in the tobacco use domain (i.e., regular use in early life [Type 2] or across life [Type 3]) do hinder advantages over CRDs in the alcohol consumption domain (i.e., being mostly a nondrinker or occasional drinker).

Fourth, in relation to the timing of the life course in which individuals are exposed to tobacco and alcohol, we provide evidence to support that trajectories showing regular consumption in one domain but only in specific periods of life, whether early or late (like Type 2 with tobacco or Type 5 with alcohol), while maintaining little or no consumption across life in the other domain (like Type 2 with alcohol or Type 5 with tobacco), lead to lower risks of CVDs or CRDs than types indicating permanent, lifelong consumption in both domains (Type 1). In other words, while it is highly inconvenient for health, it is less harmful in terms of CVD and CRD risk to smoke or drink alcohol in specific life periods than across the whole life, but only if this is accompanied by a persistent healthy behavior in the other concurrent domain.

In Chile, a significant proportion of the population who drink alcohol do not follow a moderate pattern, but one that is excessive and episodic. Therefore, our results need to be interpreted with caution, and a distinction between individual- and population-based approaches is needed. While in the individual case, clinicians might use this evidence to discuss with their patients the potential risks and benefits of moderate alcohol drinking, from a population perspective, our results showing that regular smoking plus regular drinking over the life course is a pattern associated with higher health risks might contribute to reinforcing advocacy for the implementation of cost-effective public policies to reduce tobacco and alcohol consumption nationwide [47].

This study has limitations that are important to acknowledge when interpreting results. First, because of the nature of the measurement of both the exposure to tobacco use and alcohol consumption and the risks of CVD and CRD, the possibility of time-varying covariate confounding exists. However, MCSA does not account for these time-varying covariates, but rather allows us to reconstruct representative long-term trajectory types in the two domains of interest, and then to use these types as independent variables in regression models predicting CVD and CRD risks. Second, MCSA does not account for population-level time-varying covariates either, such as anti-smoking or anti-alcohol public interventions introduced across the life course of respondents. Third, due to the multiple challenges of the autobiographical memory discussed above, the life-history calendar used in this study measured the exposure to tobacco use and alcohol consumption across the life course based exclusively on frequency questions and did not include number, amount, or types of cigarettes and alcoholic drinks. This might have influenced the power, precision, and certainty of the study findings.

## 5. Conclusions

The main results of this exploratory research are threefold. First, persistent smokers and drinkers across the life course have the highest CVD and CRD risks in old age. Second, lifetime non-smokers and non-drinkers are not ineludibly associated with low risks of CVD and CRD if they also face health risk factors such as obesity or social disadvantages such as low education. Finally, trajectories that show regular consumption in one domain but only in specific periods of life, either early or late, while having little or no consumption throughout life in the other domain, lead to lower risks of CVD or CRD, than the trajectories that indicate permanent consumption in both tobacco and alcohol domains.

The study findings have important implications for both individual and population strategies to prevent CVDs and CRDs.

In terms of individual strategies, these results might be useful for health professionals in discussing with patients the benefits of a life-course free of tobacco and alcohol. These findings can also contribute to improving cardiovascular and chronic respiratory health policies that take into account the fact that CVDs and CRDs are conditions that strongly depend on previous individual experiences in multiple life domains from infancy until old age.

In particular, population-wide interventions that affect price, marketing, and physical availability are among the most cost-effective measures to control both tobacco and alcohol consumption in the general population and have been listed by the World Health Organization as among the “best buys” for middle- and low-income countries. A life course approach can contribute to the improved design and evaluation of preventive strategies that affect tobacco and alcohol consumption across the life span.

## Figures and Tables

**Table 1:** Weighted univariate descriptive statistics.

Variable		%	Variable		%
CVD	Presence	17.34	Body mass index	Obese	30.20
	Absence	82.65		Not obese	69.80
CRD	Presence	6.16	Physical activity	1 or less a week	35.97
	Absence	93.84		>1 a week	64.03
Gender	Women	56.77	Portion of fruits or vegetables	Every day	78.91
	Men	43.23		<6 times a week	21.09
Education	Primary or none	38.51		Mean (SD)	Min–Max
	Secondary	38.89	Age	69.83 (3.14)	65–75
	Tertiary	22.60			

Note: N = 802, SD = Standard Deviation.

**Table 2:** Weighted distribution of CVD, CRD, education, gender, body mass index, physical activity, and frequency of fruits and vegetables by trajectory type.

	CVD	CRD	Education (%)			Gender (%)		Body Mass Index (%)		Physical Activity (%)		Portion of Fruits or Vegetables (%)		
Tobacco and Alcohol Trajectories Types			Primary or None	Secon Dary	Tertiary	Women	Men	Obese	Not Obese	1 or Less a Week	>1 a Week	<6 Times a Week	Every Day	Total
1. Regular smoker and drinker	53.1	12.5	13.0	47.5	39.5	12.6	87.4	6.5	93.5	45.6	54.4	40.2	59.7	10.9
2. Early-life smoker, occasional drinker	5.6	11.2	39.2	36.1	24.7	53.9	46.1	31.7	68.3	47.8	52.2	21.9	78.1	13.5
3. Regular smoker, mostly nondrinker	11.7	6.8	59.7	29.4	10.9	52.6	47.4	19.1	80.9	71.8	28.2	30.9	69.1	6.1
4. Occasional smoker, nondrinker	9.9	5.0	22.4	53.4	24.2	80.0	20.0	14.9	85.1	80.9	19.1	14.1	85.9	2.7
5. Mostly nonsmoker, early or late drinker	4.0	3.1	25.0	30.3	44.7	18.8	81.2	19.0	81.0	41.1	58.9	4.9	95.1	7.1
6. Mostly nonsmoker, occasional drinker	20.3	3.3	37.8	45.3	16.9	60.0	40.0	31.0	69.0	55.7	33.3	17.4	82.6	26.8
7. Nonsmoker, nondrinker	12.4	4.9	47.6	34.5	18.0	76.7	23.3	43.0	57.0	63.2	36.8	19.7	80.3	32.8
**Total**	17.3	6.2	38.5	38.9	22.6	56.7	43.3	30.3	69.7	56.6	43.4	21.1	78.9	100.0

Note: N = 802.

**Table 3:** Weighted logistic regression models over the probability of experiencing CVD and CRD.

	CVD			CRD		
	RRR	CI 95%	AME	RRR	CI 95%	AME
**Tobacco and alcohol trajectories types**						
(ref = Type 1. Regular smoker and drinker)						
2. Early-life smoker, occasional drinker	0.06 ***	(0.01–0.24)	–0.44	0.60	(0.11–3.06)	−0.06
3. Regular smoker, mostly nondrinker	0.13 *	(0.03–0.65)	–0.38	0.28	(0.05–1.58)	−0.12
4. Occasional smoker, nondrinker	0.13 *	(0.02–0.86)	–0.38	0.21 *	(0.05–0.85)	−0.14
5. Mostly nonsmoker, early or late drinker	0.03 **	(0.00–0.24)	–0.47	0.19 ^+^	(0.03–1.14)	−0.14
6. Mostly nonsmoker, occasional drinker	0.24 ^+^	(0.06–1.08)	–0.30	0.14 *	(0.03–0.66)	−0.15
7. Nonsmoker, nondrinker	0.12 **	(0.03–0.46)	–0.39	0.17 *	(0.04–0.87)	−0.14
**Socio-demographics**						
Age	1.08	(0.98–1.20)		1.08	(0.93–1.26)	
Gender (ref = Men)						
Women	0.70	(0.28–1.75)		2.23 ^+^	(0.90–5.54)	
Educational level (ref = Primary or none)						
Secondary	0.72	(0.34–1.56)		0.93	(0.41–2.08)	
Tertiary	1.06	(0.35–3.24)		0.28 *	(0.09–0.89)	
**Health risks**						
Body mass index (ref = Not Obese)						
Obese	1.06 *	(1.00–1.12)		0.93	(0.86–1.01)	
Physical activity (ref = More than once a week)						
Once a week or less	0.80	(0.35–1.85)		0.77	(0.30–1.93)	
Frequency fruits and vegetables (ref = Six times a week or less)						
Every day	0.80	(0.37–1.72)		1.13	(0.51–2.48)	
Intercept	0.00			0.00		

Note: Significance levels = ^+^ *p* < 0.10, * *p* < 0.05, ** *p* < 0.01, *** *p* < 0.001. RRR = Relative risk ratios. CI = Confidence Intervals. AME = Average Marginal Effects. For simplicity, only AME of tobacco and alcohol trajectory types are shown. N = 802.

## Data Availability

The data presented in this study are available on request from the corresponding author.

## Appendix A

In this study we use data from the ‘Life course and vulnerability among older people in Santiago, Chile’ study, a population-representative and longitudinal-retrospective survey. This is the first survey in Chile aiming to collect retrospective annual information on multiple dimensions of the life course, such as behavioral risk factors, coresidential histories, educational and occupational trajectories, lifetime marital and fertility patterns, work and financial vulnerabilities, and health status in old age. This representative survey collected life-course information for 802 people born between 1944–1954 (currently aged 65–75) living in Santiago, Chile’s capital city which represents approximately 40.5% of Chile’s population. The survey was conducted between March and August 2019.

Data collection followed the latest quality standards of defined by the American Association for Public Opinion Research or AAPOR. A sampling frame of individuals aged 65–75 who lived in Santiago was provided by the National Institute of Statistics (Instituto Nacional de Estadística in Spanish). The sample of 802 older individuals was randomly selected in four stages: first, blocks within the 32 communes of the city; second, households within blocks; third, households within the dwelling (if there is more than one household within a dwelling), and fourth, a person aged 65–75 within the selected the selected household or dwelling. Assuming a simple random method to select the sample, a maximum variance (*p* = 0.5), and also a 95% confidence level, the total sample error is estimated to be ±3.5 points for an infinite population. Also, in order to increase the measurement quality of the survey, eight cognitive interviews and four usability tests were conducted during the pretest phase of the questionnaires to individuals from different educational backgrounds and genders. Based on the AAPOR’s response distribution rate calculations [35], the cooperation rate was 88.8%, the response rate was 66.5%, and the refusal rate was 8.3%.

To be certain that our results were not affected by potential sample selection bias, the study sample was weighted to provide representative results for older adults aged 65–75 in the city of Santiago. Specifically, we weighted the sample by an expansion factor that accounts first, for the selection probability of individuals considering the four stages of random selection (i.e., communes, blocks, households, and persons); second, for adjustments that were made in order to compensate for the ineligibility and unknown eligibility of selected individuals in the study; and third, for a coverage adjustment or calibration, which adjusted the sample to known population characteristics and improve the accuracy of the estimates in the survey. In this study, four population characteristics were used to calibrate the sample: (i) zones of Santiago (‘north’, ‘south’, ‘center’, ‘east’, ‘west’); (ii) age range (‘65–70’ and ‘71–75’) across the five zones of Santiago; (iii) educational level (‘primary school completed or less’ and ‘secondary school or more’) across the five zones of Santiago; and (iv) gender (‘women’ and ‘men’) across the five zones of Santiago.

As mentioned above, the process of data collection involved the use of life-history calendars [36]. Whereas self-administrated life-history calendars with clear layouts and instructions lead to reliable life course data, life-history calendars administered by a well-trained interviewer who interacts with respondents while completing their calendars has yielded the most reliable and coherent life-course information [36]. The respondent–interviewer interaction not only helps the former understand how to complete the questionnaire, but also increases the number of reported events in different life domains and stages and supports a ‘double-checking’ of the provided information by the interviewer, thereby improving the precision of answers. In this research, a life-history calendar involving interviewer–respondent interaction was administered.
